# RETRACTED: Abou-Donia et al. Using Plasma Autoantibodies of Central Nervous System Proteins to Distinguish Veterans with Gulf War Illness from Healthy and Symptomatic Controls. *Brain Sci.* 2020, *10*, 610

**DOI:** 10.3390/brainsci14080758

**Published:** 2024-07-29

**Authors:** Mohamed B. Abou-Donia, Elizabeth S. Lapadula, Maxine H. Krengel, Emily Quinn, Jessica LeClair, Joseph Massaro, Lisa A. Conboy, Efi Kokkotou, Maria Abreu, Nancy G. Klimas, Daniel D. Nguyen, Kimberly Sullivan

**Affiliations:** 1 Department of Pharmacology and Cancer Biology, Duke University Medical Center, Durham, NC 27710, USA; donia@duke.edu (M.B.A.-D.); elizabeth.lapadula@duke.edu (E.S.L.); 2 Department of Neurology, Boston University School of Medicine, Boston, MA 02118, USA; mhk@bu.edu; 3 Departments of Biostatistics and Environmental Health, Boston University School of Public Health, Boston, MA 02118, USA; eq@bu.edu (E.Q.); jleclai2@bu.edu (J.L.); jmm@bu.edu (J.M.); ddn@bu.edu (D.D.N.); 4 Department of Medicine, Harvard Medical School, Boston, MA 02115, USA; lisa_conboy@hms.harvard.edu (L.A.C.); ekokkoto@bidmc.harvard.edu (E.K.); 5 Dr. Kiran C. Patel College of Osteopathic Medicine, Institute for Neuroimmune Medicine, Nova Southeastern University, Fort Lauderdale, FL 33314, USA; mariamercedesabreu@gmail.com (M.A.); nklimas@nova.edu (N.G.K.); 6 Department of Immunology, Miami VA Medical Center, Miami, FL 33125, USA

The *Brain Sciences* Editorial Office retracts the article “Using Plasma Autoantibodies of Central Nervous System Proteins to Distinguish Veterans with Gulf War Illness from Healthy and Symptomatic Controls” [1], cited above.

Following publication, concerns were brought to the attention of the Editorial Office by a representative from Duke University regarding the validity of the Western blots presented in Figures 1–3 of this publication [1].

Adhering to our complaints procedure, an investigation was conducted by the Editorial Office and Editorial Board. Based on the review by Duke University of the article’s findings, the co-authors have requested a retraction due to concerns about their ability to rely on the Western blot images and produce verified, reliable results.

The Editorial Board evaluated the correspondence from the authors and the Institution and decided to retract this article [1], as per MDPI’s retraction policy (https://www.mdpi.com/ethics#_bookmark30, accessed on 28 May 2024) and in line with the Committee on Publication Ethics retraction guidelines (https://publicationethics.org/retraction-guidelines, accessed on 28 May 2024).

This retraction was approved by the Editor-in-Chief of the journal *Brain Sciences*. 

The authors agreed to this retraction.
